# Major risk factors underlying the development of metabolic syndrome in vitamin D-deficient rats

**DOI:** 10.3389/fphar.2025.1573332

**Published:** 2025-04-28

**Authors:** Fatemah O. Kamel, S. K. Mahjoub, M. A. A. Sattar Ahmad, Maha H. Jamal, Duaa M. Bakhshwin, Abdulhadi S. Burzangi, Soad Shaker, Rania Magadmi

**Affiliations:** ^1^ Department of Clinical Pharmacology, Faculty of Medicine, King Abdulaziz University, Jeddah, Saudi Arabia; ^2^ Anatomy Department, Faculty of Medicine, King Abdulaziz University, Jeddah, Saudi Arabia

**Keywords:** cardiovascular complications, metabolic syndrome, oxidative stress, vascular function, vitamin D deficiency

## Abstract

**Background:**

Vitamin D is essential for calcium–phosphorus homeostasis, skeletal mineralization, and cardiovascular health. Its deficiency is associated with increased risk of metabolic syndrome and cardiovascular diseases. In this study, we aimed to investigate the relationship between vitamin D deficiency and cardiovascular metabolic syndrome while identifying underlying mechanisms.

**Methodology:**

Forty-eight Wistar albino rats were divided into four groups: control, vitamin D deficient (VD-), metabolic syndrome (MetS), and combined vitamin D deficient with metabolic syndrome (VD- + MetS). VD- and VD- + MetS rats were fed a vitamin D-deficient diet with increased calcium and phosphate to prevent secondary hyperparathyroidism and to determine the direct effects of vitamin D. Metabolic syndrome was induced *via* 10% fructose in drinking water for 8 weeks. Evaluations included metabolic syndrome markers (hypertension, diabetes, dyslipidemia, and obesity), myocardial injury indicators (lactate dehydrogenase [LDH] and creatine kinase-MB [CK-MB]), and oxidative stress/inflammation markers (malondialdehyde [MDA] and nitric oxide [NO]). Vascular reactivity in thoracic aorta tissues, heart weight, and histopathological changes were also assessed.

**Result:**

The results revealed that vitamin D deficiency was strongly related to each component of metabolic syndrome. Combined vitamin D deficiency and metabolic syndrome induced a highly significant increase in CK-MB, LDH, NO, and MDA levels (p < 0.05). However, there was no significant difference in CK-MB and NO levels for the (VD-) group compared to the control (p > 0.05). Heart weight was significantly increased, and a histological examination of the heart showed increased left ventricular and aortic wall thickness in the combined group (p < 0.05). Vascular response to phenylephrine was significantly increased, whereas the vascular response to acetylcholine was significantly decreased in all experimental groups (VD-, MetS, and VD- + MetS) compared to control (p < 0.05).

**Conclusion:**

The present study demonstrates that vitamin D deficiency is considered one of the major risky and predisposing factors for cardiovascular metabolic syndrome, which could affect the outcome of the disease, partly by affecting endothelial function, vascular oxidative stress, and inflammation.

## 1 Introduction

Vitamin D is essential for several critical physiological processes of the body, including facilitating the intestinal absorption and renal reabsorption of calcium, maintenance of calcium–phosphorus homeostasis, regulating the parathyroid hormone (PTH), and mediating skeletal mineralization and bone formation (Kulie et al., 2009; Mandarino et al., 2015). The biologically active form of vitamin D, that is, calcitriol (1,25-dihydroxycholecalciferol), serves as a key regulator of calcium and phosphorus balance, acting as a potent ligand for the vitamin D receptor (VDR), which mediates many of its physiological functions (Wang, 2013). VDRs are widely distributed across various tissues, including the vascular smooth muscle, myocardium, and endothelium, underscoring the importance of vitamin D in cardiovascular health (2–4). It modulates the immune system by enhancing antimicrobial peptides and regulating T-cell responses, thus reducing the risk of infections and autoimmune diseases (Prietl et al., 2013). Vitamin D also has anti-inflammatory effects, lowering pro-inflammatory cytokines and benefiting chronic conditions such as cardiovascular disease (Guillot et al., 2010). Additionally, it regulates insulin secretion and sensitivity, impacting diabetes (Norman et al., 1989). Vitamin D’s neuroprotective role is evident through its association with reduced cognitive decline (Annweiler et al., 2013), making it crucial for overall health.

Vitamin D improves endothelial function by enhancing nitric oxide (NO) synthesis, reducing oxidative stress, and preventing endothelial dysfunction, which is crucial in atherosclerosis and hypertension (Kienreich et al., 2013). Additionally, vitamin D inhibits the renin–angiotensin–aldosterone system (RAAS), reducing arterial stiffness and hypertension (Al Mheid et al., 2013). It also prevents vascular calcification and myocardial hypertrophy by downregulating pro-inflammatory cytokines such as TNF-α (Lee et al., 2008). Vitamin D deficiency is linked to left ventricular dysfunction and heart failure, highlighting its importance in maintaining cardiac function (Pilz et al., 2011). Recent studies further elucidate the pivotal role of vitamin D in cardiovascular diseases, showing its influence on endothelial function, vascular inflammation, and cardiomyocyte health (Islam et al., 2024). Vitamin D deficiency is strongly associated with cardiometabolic disorders, namely, insulin resistance, dyslipidemia, and inflammation. Vitamin D modulates insulin secretion by binding to VDRs in pancreatic beta cells, improving glucose metabolism and reducing insulin resistance (Pittas et al., 2007). It also affects the lipid metabolism by reducing triglycerides and improving HDL cholesterol levels (Vacek et al., 2012). Moreover, vitamin D downregulates inflammatory markers such as C-reactive protein (CRP) and TNF-α, reducing systemic inflammation linked to metabolic syndrome (Deleskog et al., 2012).

The prevalence of vitamin D deficiency has become a growing public health concern worldwide, particularly in regions such as Saudi Arabia, where traditional clothing, limited sun exposure, and insufficient dietary intake contribute to widespread deficiency (Ardawi et al., 2012). Vitamin D insufficiency in the United States showed an increased prevalence of up to 90% of the black, Asian, and Hispanic populations and three-quarters of the Caucasian population in the last 10 years (Mandarino et al., 2015). A severe deficient level of vitamin D (≤10 ng/ml) is found in most of the Asian and Middle East populations (Alsuwaida et al., 2013) and even in Saudi Arabia. A study that was carried out in Riyadh, seeking the prevalence of vitamin D deficiency among 488 healthy adults, has shown that 29% of the subjects were vitamin D deficient, whereas 22.7% were relatively insufficient (Ardawi et al., 2012). Another cross-sectional study that was carried out on 834 randomly selected Saudi men aged between 20–74 years living in Jeddah had revealed that the prevalence of vitamin D deficiency was 87.8% among Saudi men (Kato, 2000).

Several studies showed that there was a strong association between vitamin D deficiency and cardiometabolic risk and other disorders such as hypertension, atherosclerosis, dyslipidemia, diabetes, and obesity (Hlyan et al., 2024; Levin and Li, 2005; Mandarino et al., 2015; Mithal et al., 2009). Despite growing evidence, the precise mechanisms remain unclear, especially in specific populations such as those in Saudi Arabia, where the lifestyle and environmental factors play a significant role. Therefore, the present study was directed to study the link between vitamin D deficiency and the development of cardiovascular metabolic syndrome, and further clarify the underlying mechanisms. The aims of this study was to address this research gap by investigating the effects of vitamin D deficiency on cardiometabolic syndrome. Moreover, the deleterious effect of combined vitamin D deficiency and metabolic syndrome was studied. Furthermore, the mechanisms underlying this effect were also investigated.

## 2 Materials and methods

### 2.1 Experimental design

A total of 48 adult male Wistar albino rats, weighing between 140 and 220 g, were procured from the King Fahad Research Center at King Abdulaziz University, Jeddah, Saudi Arabia. The rats were housed individually in standard cages under controlled environmental conditions with a 12-h light–dark cycle. They were acclimatized for 1 week prior to the experimental procedures and had *ad libitum* access to food and water. The study adhered to the ethical guidelines of the King Fahad Research Center, with prior approval from the Institutional Ethical Committee (Reference No. 148–19).

The rats were divided into four distinct groups (12/each group) based on their dietary and treatment regimens. The control group (C) received a regular diet and water *ad libitum*. The vitamin D-deficient group (VD-) was fed a vitamin D-deficient diet (Harlan Teklad Custom Diet TD.87095) from the start of the study. Additionally, these rats were administered intraperitoneal injections of paricalcitol (32 ng) on days 1, 3, 5, 8, 10, and 12 to induce vitamin D deficiency by enhancing the catabolism of endogenous vitamin D metabolites. The metabolic syndrome group (MetS) was fed a regular diet supplemented with 10% fructose in their drinking water for 8 weeks to induce metabolic syndrome. Finally, the combined group (VD- + MetS) was subjected to both vitamin D deficiency and metabolic syndrome by following the same protocols as the VD- and MetS groups, respectively, maintaining the vitamin D deficiency diet and 10% fructose water for 8 weeks. The diets of the vitamin D-deficient groups (VD- and VD- + MetS) included increased calcium and phosphate levels to prevent secondary hyperparathyroidism.

Two types of diets were followed: one was the vitamin D-deficient diet, that is, Harlan Teklad Custom Diet (TD 87095), from Envigo^®^, USA. This diet, which is deficient in vitamin D, contained 20% lactose, 15% corn starch, 17.6% protein, 5% fat, 2% calcium, and 1.25% phosphate. The other was the regular diet, which was obtained from the Grain Silos & Flour Mills Organization, KSA. This diet contained 20% protein, 4% fat, 1% calcium, 0.6% phosphate, and 2.20 IU/g vitamin D.

Body weights were recorded at baseline and after 8 weeks using a calibrated electronic scale. The weight gain percentage was calculated as the difference between the final and initial weights.

### 2.2 Blood sample collection

Blood samples were collected under light ether anesthesia from the retro-orbital venous plexus using capillary tubes and stored in 5 ml tubes. The samples were centrifuged at 4,500 rpm for 15 min. Blood collection occurred at baseline, week 3 (to confirm vitamin D deficiency), and at the end of the 8-week period. Biochemical assessments for metabolic syndrome indicators such as hypertension, diabetes mellitus, dyslipidemia, and obesity were performed. Additional assays included lactate dehydrogenase (LDH), serum malondialdehyde (MDA) for oxidative stress, and NO for inflammation.

### 2.3 Cardiovascular markers

Serum lactate dehydrogenase (LDH) and creatine kinase MB (CK-MB) levels were measured at baseline and after 8 weeks. LDH activity was assessed using the Dimension Vista^®^ SIEMENS system by monitoring NADH formation at 340 nm. CK-MB levels were determined with the HUMAN^®^ kit, measuring enzymatic activity and reporting results in U/L. Serum MDA levels were determined using the TBARS assay kit from Abcam^®^, with levels expressed in nmol/mL. NO was measured using the Abcam^®^ assay, reflecting total nitrate and nitrite levels, which were expressed in µM/L.

### 2.4 Aortic vascular reactivity

The tissue bath system was preheated to 37°C and connected to a 95% oxygen and 5% carbon dioxide atmosphere. The Krebs–Henseleit buffer was aerated and maintained at optimal conditions. Aortic rings were dissected, cleaned of perivascular adipose tissue and blood clots, and sectioned into 3–5 mm rings. Each ring was suspended in the tissue bath, equilibrated at 8 mN resting tension, and exposed to incremental tension to assess vascular reactivity.

### 2.5 Assessment of cardiac hypertrophy

#### 2.5.1 Heart weight measurement

Rats were weighed and then killed by cervical dislocation. The chest cavity was then rapidly opened to remove the heart for blotting dry for weighing. The heart weight was calculated relative to the body weight at the time when the organ was removed and expressed in (mg/g). This was performed using a sensitive weighing instrument.

#### 2.5.2 Histopathological examination

The animals were anesthetized with ether and then killed by cervical dislocation. The chest was opened, and intra-thoracic organs were rinsed by saline followed by phosphate buffer to wash blood. Heart and thoracic aorta were dissected out.

Aorta was cut at the end of the aortic arch; spread on card paper; cut into proximal, middle, and distal parts; fixed in 10% neutral buffered formalin; and processed using an automatic paraffin processing machine (dehydration by ascending grades of alcohol, xylene, paraffin wax, and blocking). Then, 5-micron sections from each part was stained by hematoxylin and eosin stain (H&E) for the general structure and morphometric study. Masson trichrome stain was used for collagen according to Bancroft and Layton (2018).

For the heart histological examination, cross-sections were taken 1 cm from the apex and processed similarly to the aorta. Slides were photographed using an Olympus Microscope BX-51 at a magnification of (×4), which is high resolution that ensured better observation of the specimen with a digital camera connected to a computer (Pandurić et al., 2012).

#### 2.5.3 Morphometric study

The intima media thickness was measured in all graphs in three different parts of the aorta and performed on photographs taken with a 10, 40 objective lens and a 10-ocular lens (Olympus digital camera) using special software (Pro Plus image analysis software version 6.0.). Six fields of three sections from each animal were used for this step. Photographs for the cardiac walls were analyzed for any left ventricular thickness using the same software (Pandurić et al., 2012).

### 2.6 Statistical analysis

Biochemical parameters, blood pressure, histopathological screening, vascular reactivity, heart weight, body weight increase %, BMI< adiposity index, abdominal fat weight, and AC were analyzed. All biochemical results were presented as mean ± SD. The mean change in biochemical measures in the experimental group from 0 to 3 and 8 weeks was assessed using a paired t-test. One-way ANOVA followed by Tukey HSD *post hoc* test was used to compare the groups using GraphPad Prism and Excel 2016. The p-value <0.05 was considered statistically significant.

## 3 Results

### 3.1 Assessment of cardiovascular biomarkers

**FIGURE 1:**
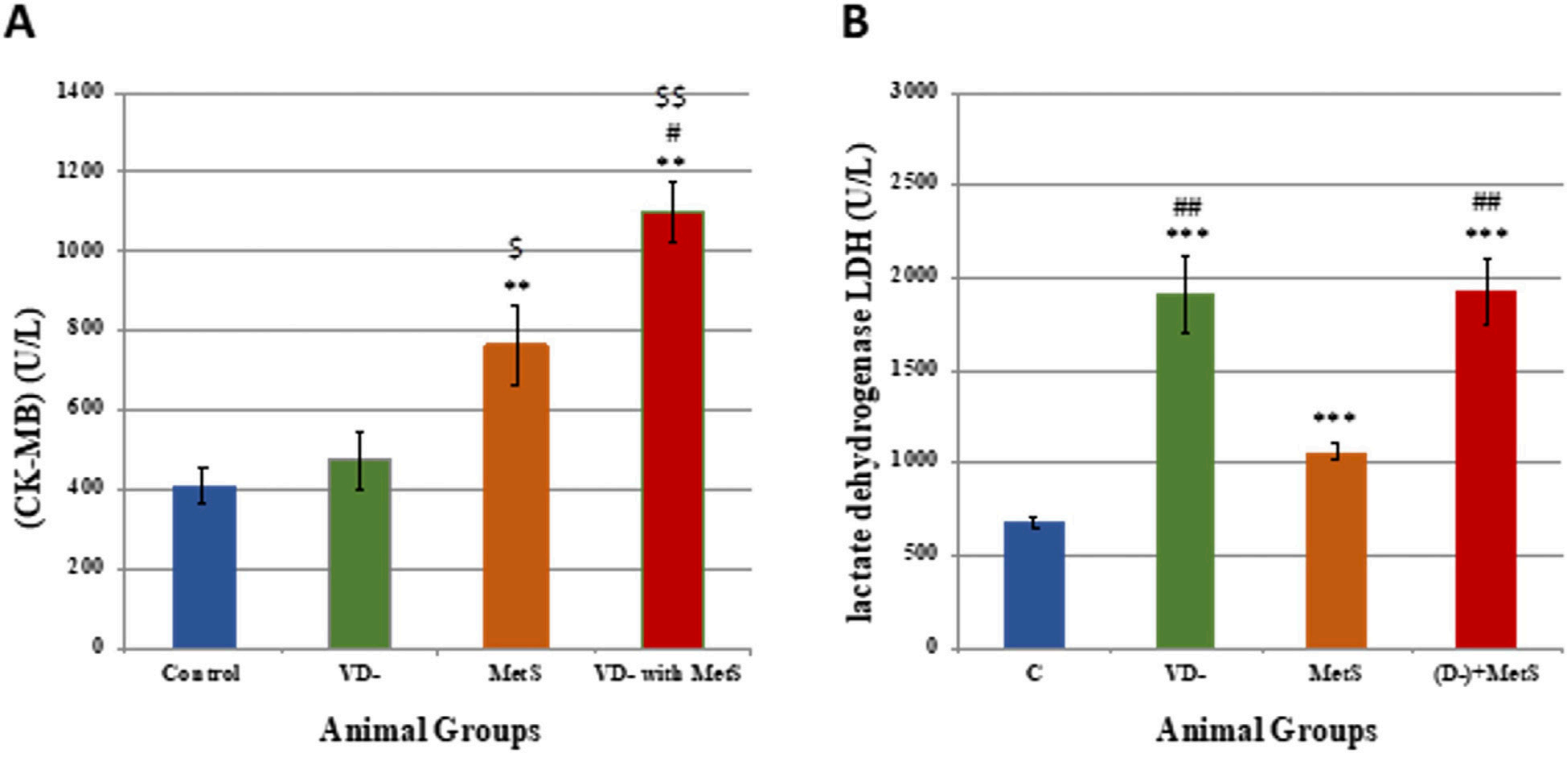
Assessment of cardiovascular markers levels for control, VD-, MetS, and (VD- + MetS) groups after 8 weeks (n = 12 rat/group). **(A)** Mean ± S.E of serum creatine kinase MB level. **(B)** Mean ± S.E of serum lactate dehydrogenase level. VD-: vitamin D-deficient group, MetS: metabolic syndrome group, and (VD- + MetS): combined group (vitamin D-deficient and metabolic syndrome). *** significant (p < 0.0001) vs control group. ## significant (p < 0.001) vs. MetS group.

Figure 1A shows a significant increase in the CK-MB level for the metabolic syndrome (MetS) group (p < 0.001) and the combined (VD- + MetS) group (p < 0.001) compared to the control, whereas the vitamin D-deficient (VD-) group showed nonsignificant increase (p > 0.05) compared to the control. Furthermore, a significant (p < 0.0001) increase in the LDH level was seen with all test groups (VD-, MetS, and (VD- + MetS) compared to the control group. Interestingly, the vitamin D-deficient (VD-) group showed a significant increase in the LDH level (p < 0.001) compared to the metabolic syndrome (MetS) group (Figure 1B).

### 3.2 Assessment of oxidative stress

#### 3.2.1 Malondialdehyde (MDA) serum level

**FIGURE 2:**
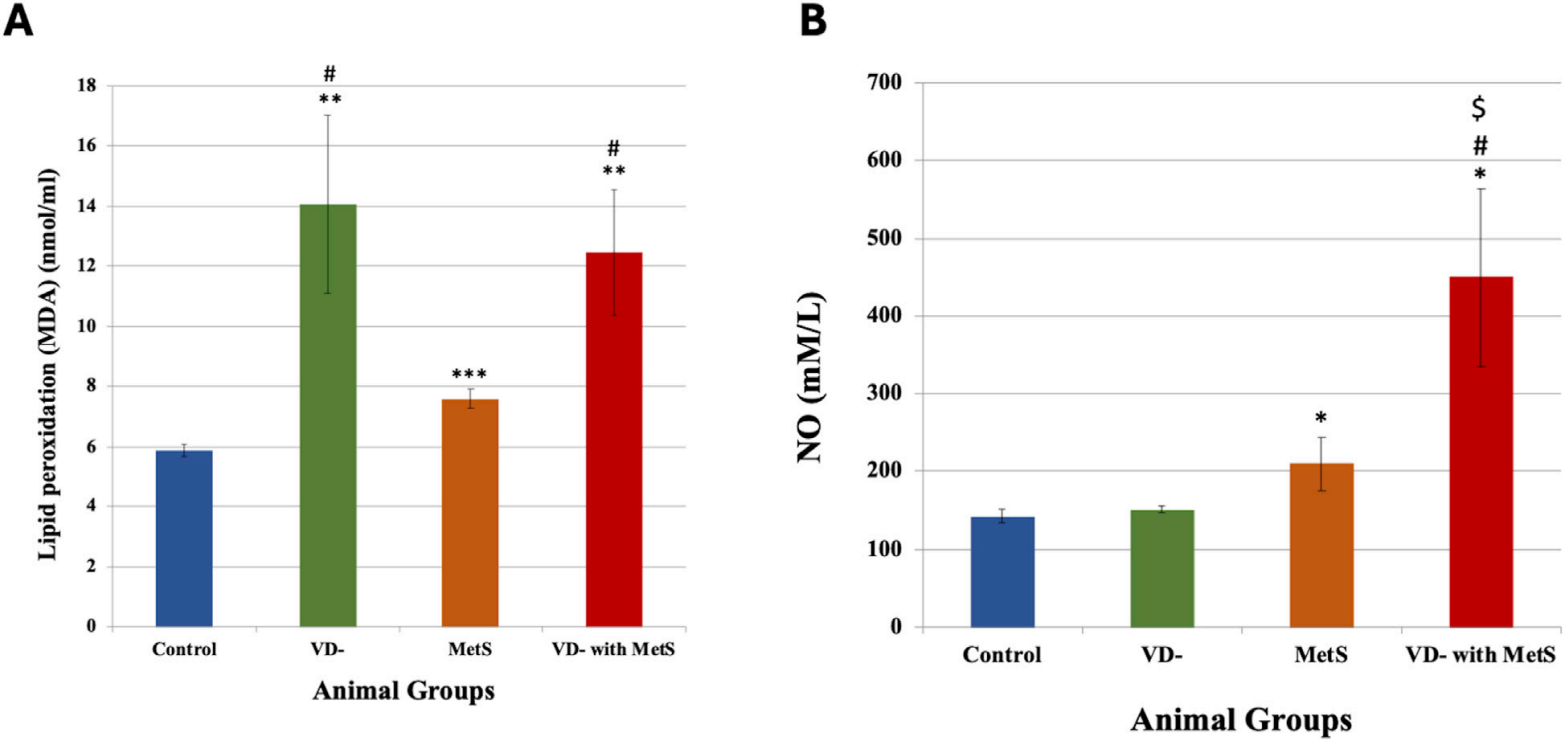
Serum malondialdehyde level **(A)** and nitric oxide level **(B)** for control, VD-, MetS, and (VD- + MetS) groups after 8 weeks (n = 12 rat/group). Mean ± SD. VD-: vitamin D-deficient group, MetS: metabolic syndrome group, and (VD- + MetS): combined group (vitamin D-deficient and metabolic syndrome). *significant (p < 0.05) vs control group, # significant (p < 0.05) vs MetS group, $ significant (p < 0.05) vs. VD- group.

It is obvious from Figure 2A that the MDA level was significantly increased in the vitamin D-deficient (VD-) group (p < 0.001), metabolic syndrome (MetS) group (p < 0.0001), and combined (VD- + MetS) group (p < 0.001) compared to the control group. In addition, the vitamin D-deficient (VD-) and the combined (VD- + MetS) groups produced a significant increase in the MDA level (p < 0.01) compared to the metabolic syndrome (MetS) group.

#### 3.2.2 Nitric oxide (NO) level

Figure 2B showed a significant increase in the NO level in the combined (VD- + MetS) group (p < 0.05) and the metabolic syndrome (MetS) group (p < 0.05) compared to the control group. A nonsignificant (p > 0.05) increase was observed with the vitamin D-deficient (VD-) group compared to the control group.

### 3.3 Effect on vascular reactivity

#### 3.3.1 Response to vasopressors

**FIGURE 3:**
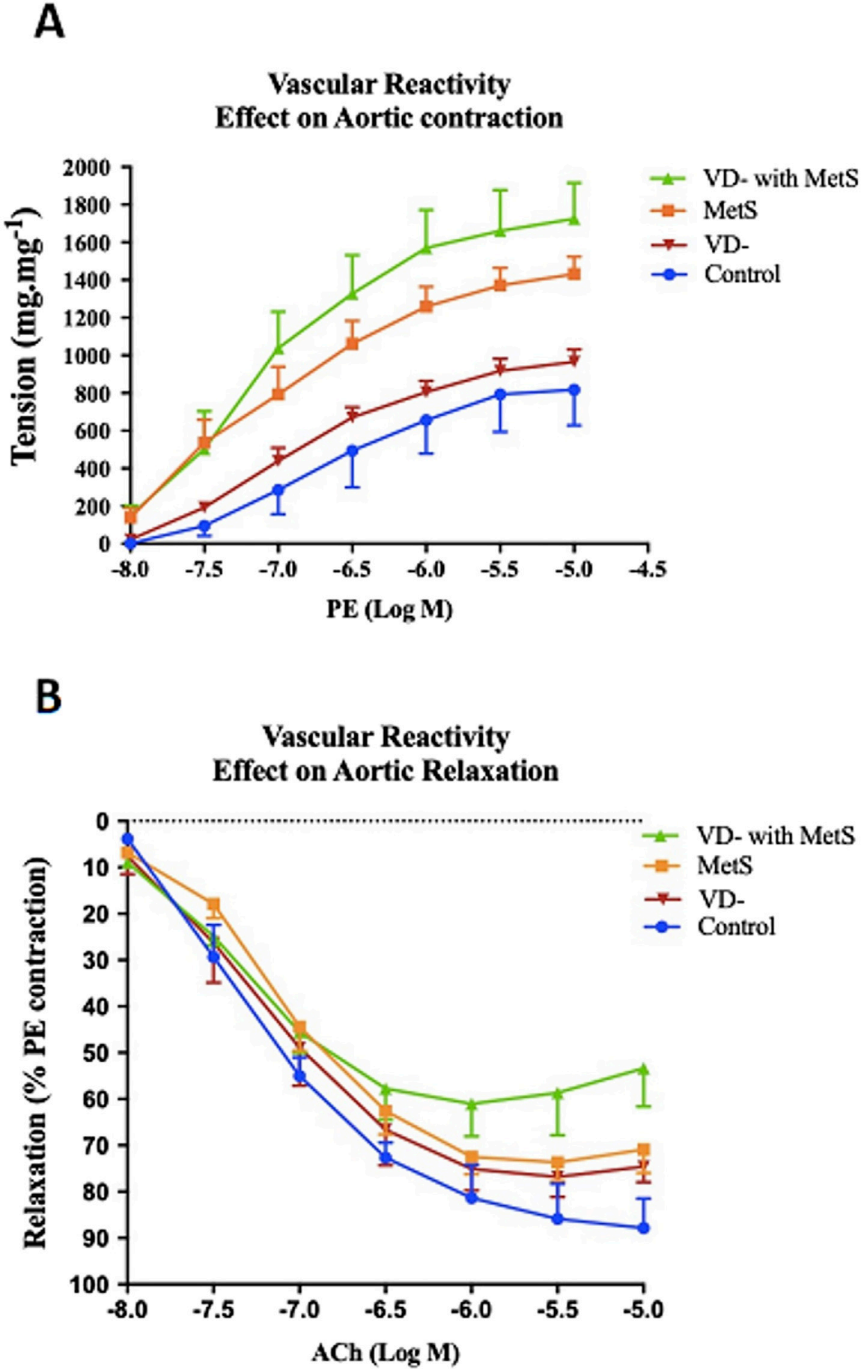
Effect of vitamin D deficiency and/or metabolic syndrome on isolated aorta responsiveness to **(A)** phenylephrine (PE) and **(B)** acetylcholine (Ach) after 8 weeks (n = 12 rat/group).

As seen from Figure 3A, adding phenylephrine PE (10-8- 10–5 M) to the organ bath produced concentration-dependent contraction of the aorta in all groups. The vitamin D-deficient (VD-), metabolic syndrome (MetS), and combined (VD- + MetS) groups showed a significantly large increase in aortic responsiveness to PE (p < 0.0001). Figure 3B showed that adding cumulative concentrations of acetylcholine Ach (10-8–10–5 M) to the organ bath resulted in concentration-related decreases in the aortic ring tension pre-contracted with Ach. All test groups resulted in a significant decrease in aortic responsiveness to Ach in the following order: vitamin D deficiency (VD-) group (p < 0.01), metabolic syndrome (MetS) group (p < 0.0001), and combined (VD- + MetS) group (p < 0.0001).

### 3.4 Effect on heart morphology and histology

#### 3.4.1 Heart weight measurement

Heart weight relative to body weight was significantly increased in the vitamin D-deficient (VD-), metabolic syndrome (MetS), and combined (VD- + MetS) groups compared to the control (p < 0.05). However, no significant difference was observed between the VD- and MetS groups or between the combined (VD- + MetS) group and either the VD- or MetS groups (p > 0.05) (Table 1).

**TABLE 1:** Mean value of the heart weight relative to the body weight after 8 weeks.

Parameters	Control	Vitamin D deficient (VD-)	Metabolic syndrome (MetS)	Combined (VD-) + (MetS)
Heart weight relative to body weight (mg/g)	3.4 ± 0.1	4.4 ± 0.2^a^	4.0 ± 0.20^a^	4.2 ± 0.12^a^

Number of rats per group = 12.

Values are expressed as the means ± S.E.

^a^ Significant (p < 0.05) vs control group.

#### 3.4.2 Morphometric study of the heart (left ventricle thickness)

**FIGURE 4:**
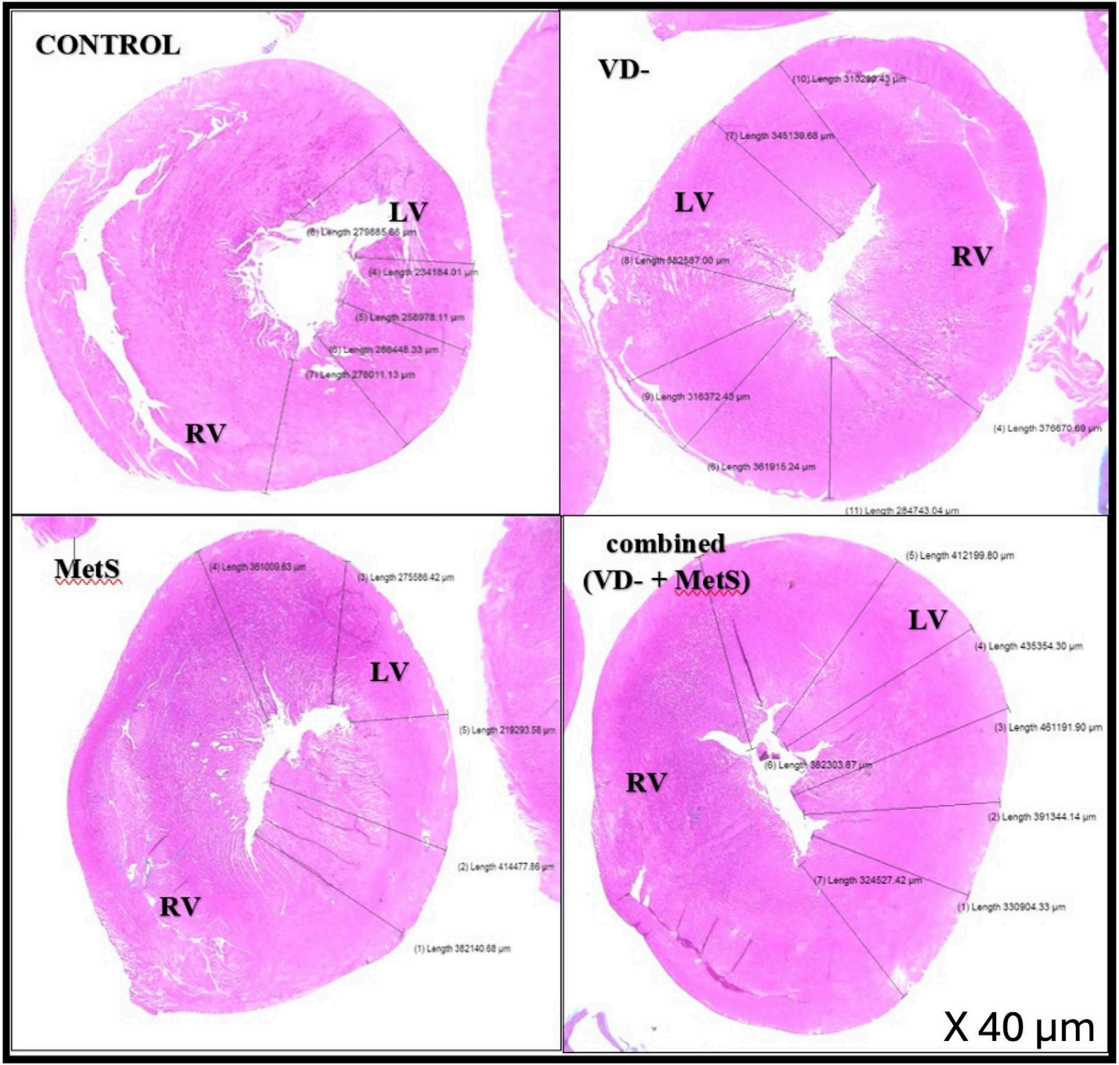
Cross-sections in cardiac wall stained by H&E showing relative thickness of the left ventricular wall for control, VD-, MetS, and combined (VD- + MetS) groups.

Figure 4 displays histological sections of the left ventricular wall, stained with hematoxylin and eosin (H&E), across the control, vitamin D-deficient (VD-), metabolic syndrome (MetS), and combined (VD- + MetS) groups. The cross-sectional images vividly demonstrate the progressive thickening of the left ventricular wall, with clear variations in structural organization among the groups. In the control group, the left ventricular wall maintains normal thickness, with well-organized muscle fibers, indicating normal cardiac function. In the vitamin D-deficient (VD-) group, notable thickening and mild disorganization of fibers suggest early hypertrophy due to impaired calcium regulation and oxidative stress. The metabolic syndrome (MetS) group shows moderate thickening, with focal degeneration of muscle fibers, reflecting early myocardial damage from cardiometabolic disturbances. The combined VD- + MetS group exhibits the most significant thickening, with severe hypertrophy, disorganization, and atrophy, highlighting the synergistic effect of vitamin D deficiency and metabolic syndrome on cardiac remodeling and dysfunction.

**FIGURE 5:**
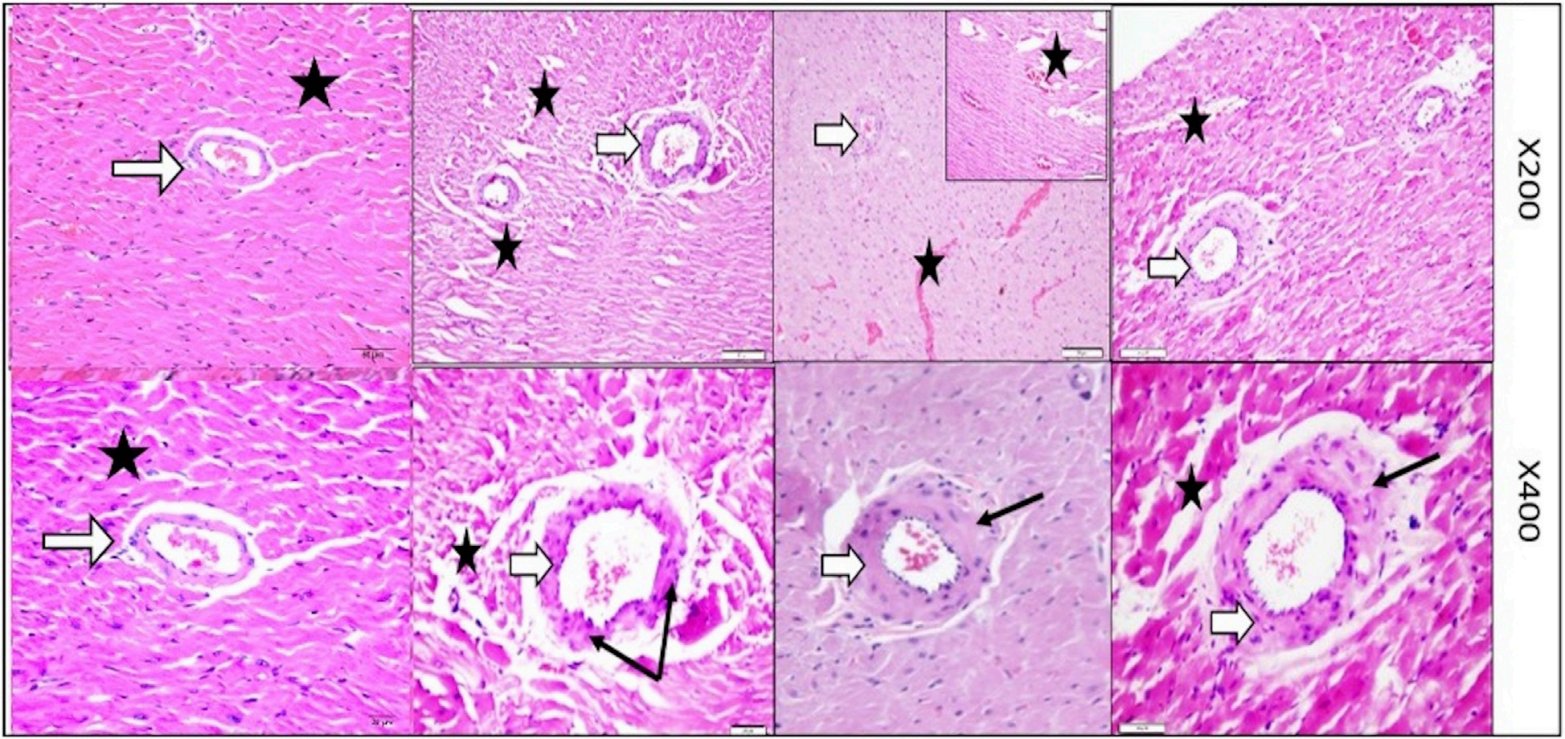
Magnified powers (×200 and ×400) of parts of rat heart left ventricle stained by H&E to show coronary artery (white arrows) and its wall thickness (black arrows). Control group with normal coronary wall thickness with normal wall thickness (white arrow) and well-organized nearby cardiac muscle fibers (star). MetS group showing mild irregular focal thickening of the coronary wall along with focal degeneration (stars). VD- group showing marked focal thickening of the coronary wall along with vascular congestion and focal muscle loss (stars). Combined (VD- + MetS) group showing more increase in coronary wall thickness with focal atrophy and destruction of cardiac muscle (stars).

Figure 5 provides magnified views (200× & 400×) of the left ventricular tissue, focusing on the coronary artery wall thickness and the organization of the surrounding cardiac muscle fibers in the control, VD-, MetS, and combined VD- + MetS groups. This figure highlights how both the coronary artery and myocardial fibers are affected by the experimental conditions. In the control group, the coronary artery wall maintains normal thickness, with organized elastic fibers, smooth muscle cells, and healthy cardiac muscle. The MetS group shows mild thickening and focal irregularity of the artery wall, with early cardiac muscle degeneration due to metabolic disturbances. In the vitamin D-deficient group, the artery wall is markedly thicker, with vascular congestion and disorganized muscle fibers, indicating oxidative stress and impaired function. The combined VD- + MetS group exhibits severe artery wall thickening, vascular congestion, and extensive cardiac muscle atrophy, reflecting the compounded impact of both conditions on coronary blood flow and heart structure.

**FIGURE 6:**
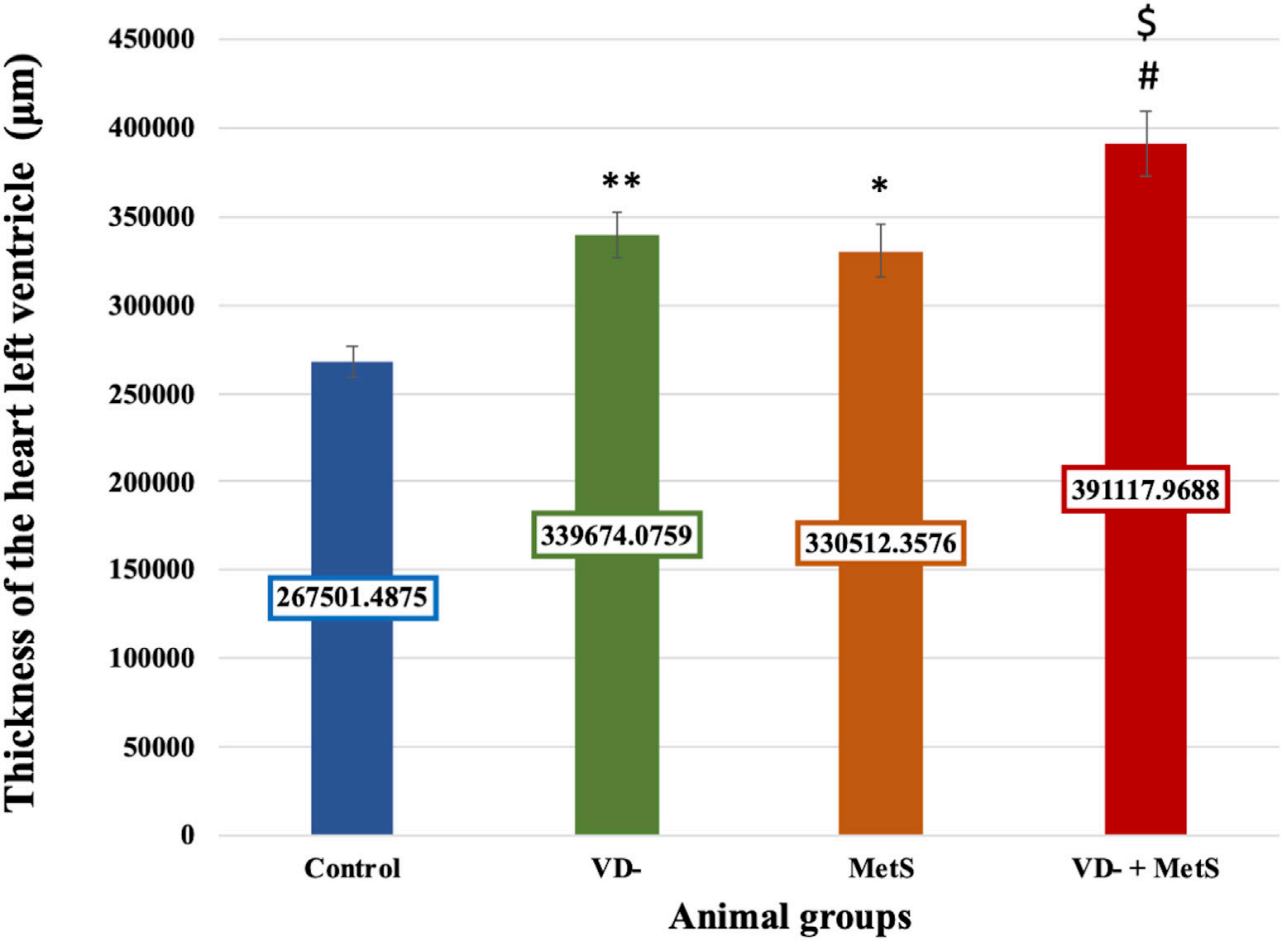
Mean ± S.E level of the thickness of heart left ventricle for control, VD-, MetS, and (VD- + MetS) groups after 8 weeks. VD-: vitamin D-deficient group, MetS: metabolic syndrome group, and VD- + MetS: combined group (vitamin D deficient and metabolic syndrome), n = 12. Mean is expressed as mean ± SE. *** significant (p < 0.0001) vs control group. ** significant (p < 0.001) vs control group. * significant (p < 0.01) vs control group. # significant (p < 0.05) vs metabolic syndrome group. $ significant (p < 0.05) vs vitamin D-deficient group.

The left ventricular thickness showed a significant rise in the VD- (p < 0.001), MetS (p < 0.01), and combined (VD- + MetS) groups (p < 0.0001) compared to the control. The combined group also exhibited a significant increase in left ventricle thickness compared to both the VD- and MetS groups (p < 0.05), as shown in Figure 6.

### 3.5 Effect on aorta morphology and histology

**FIGURE 7:**
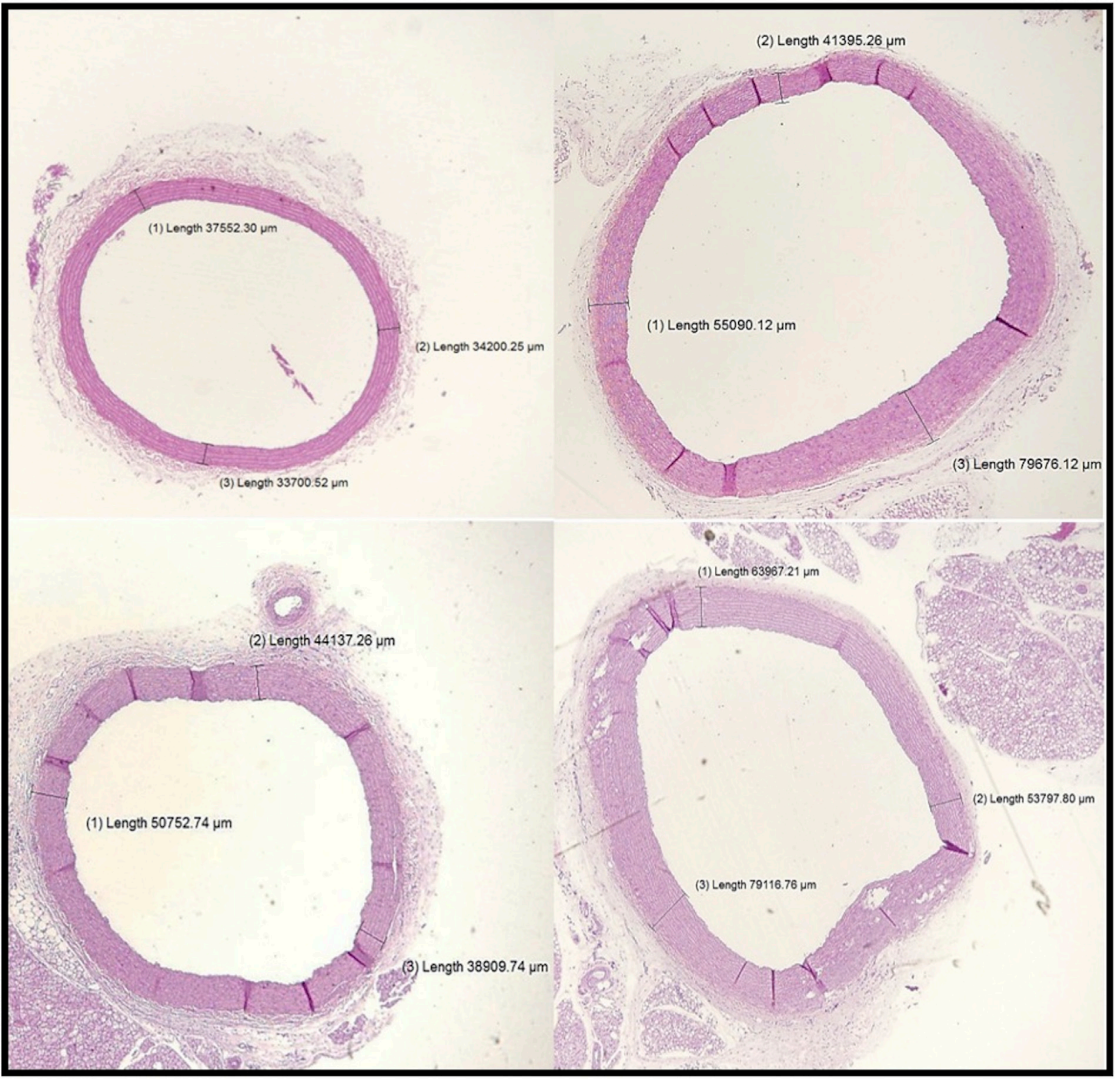
Sections of the aorta stained by H&E (200 μm) showing intima-media thickness for control, VD-, MetS, and (VD- + MetS) groups.

**FIGURE 8:**
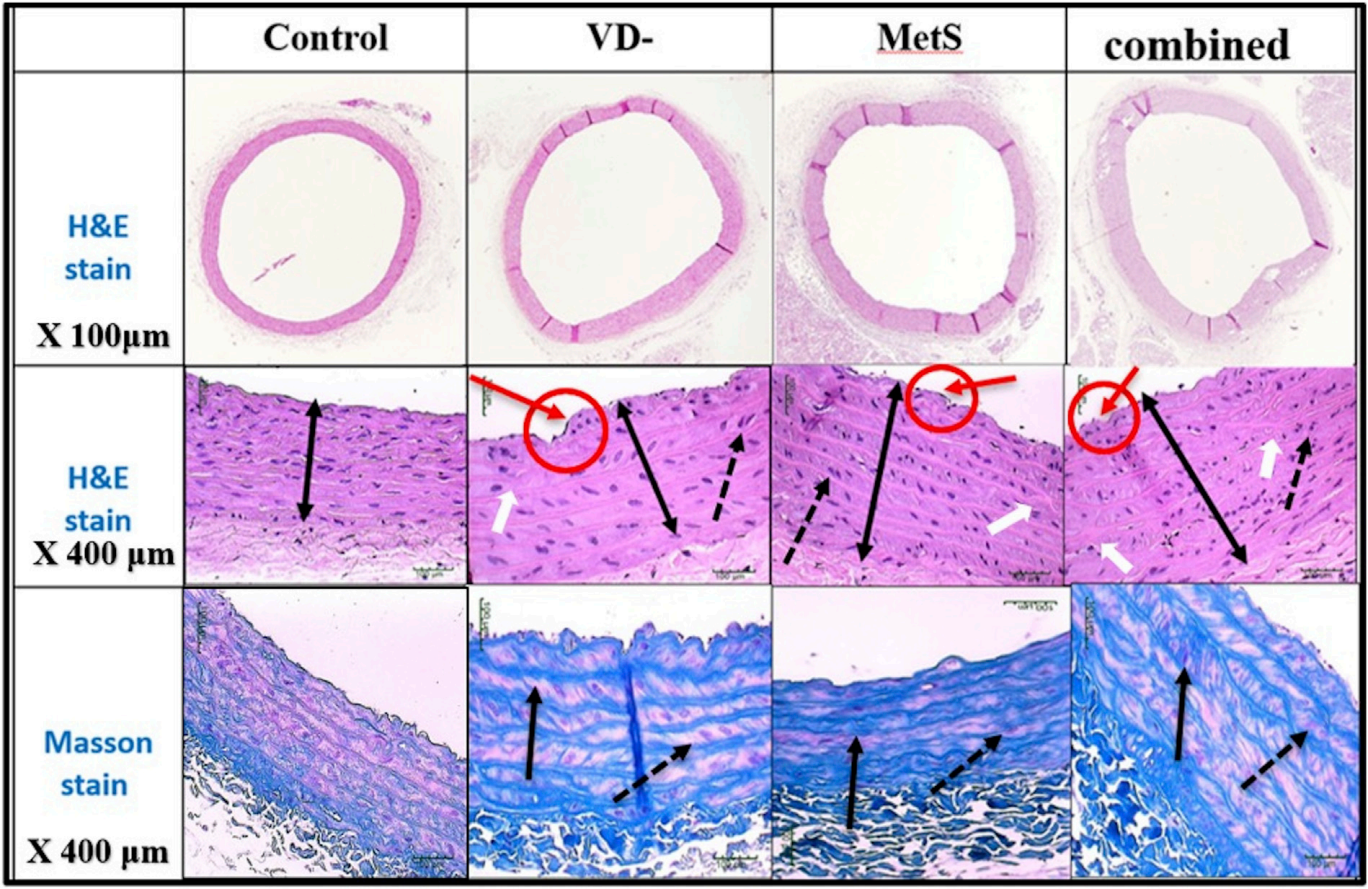
Sections of the aorta stained by H&E and Masson stain showing intima-media thickness for control, VD-, MetS, and combined (VD- + MetS) groups. The irregularity of the endothelial layer with focal thickening of the intima layer (red circle and arrows), increase in intima-media layer thickness (double heads arrow), smooth muscle in-between elastic fibers showed proliferation and vacuolation (doted black arrows), and focal irregularity and thickening of elastic fibers (white arrows). Masson stain showed thickening and irregularity of elastic fibers (black arrows) and proliferation of smooth muscles (doted arrows).

The aortic wall in the control rat was found to consist of an intima media layer as it was difficult to distinguish between the inner layer (intima) and the middle layer (media) as separate entities, and this layer mainly consisted of wavy elastic fibers that alternate with smooth muscle. Next to the intima media layer, there is a layer of loose connective tissue that mainly consisted of collagen fibers, few connective tissue cells, and tiny blood capillaries, and it is known as adventitia (Figures 7, 8).

**FIGURE 9:**
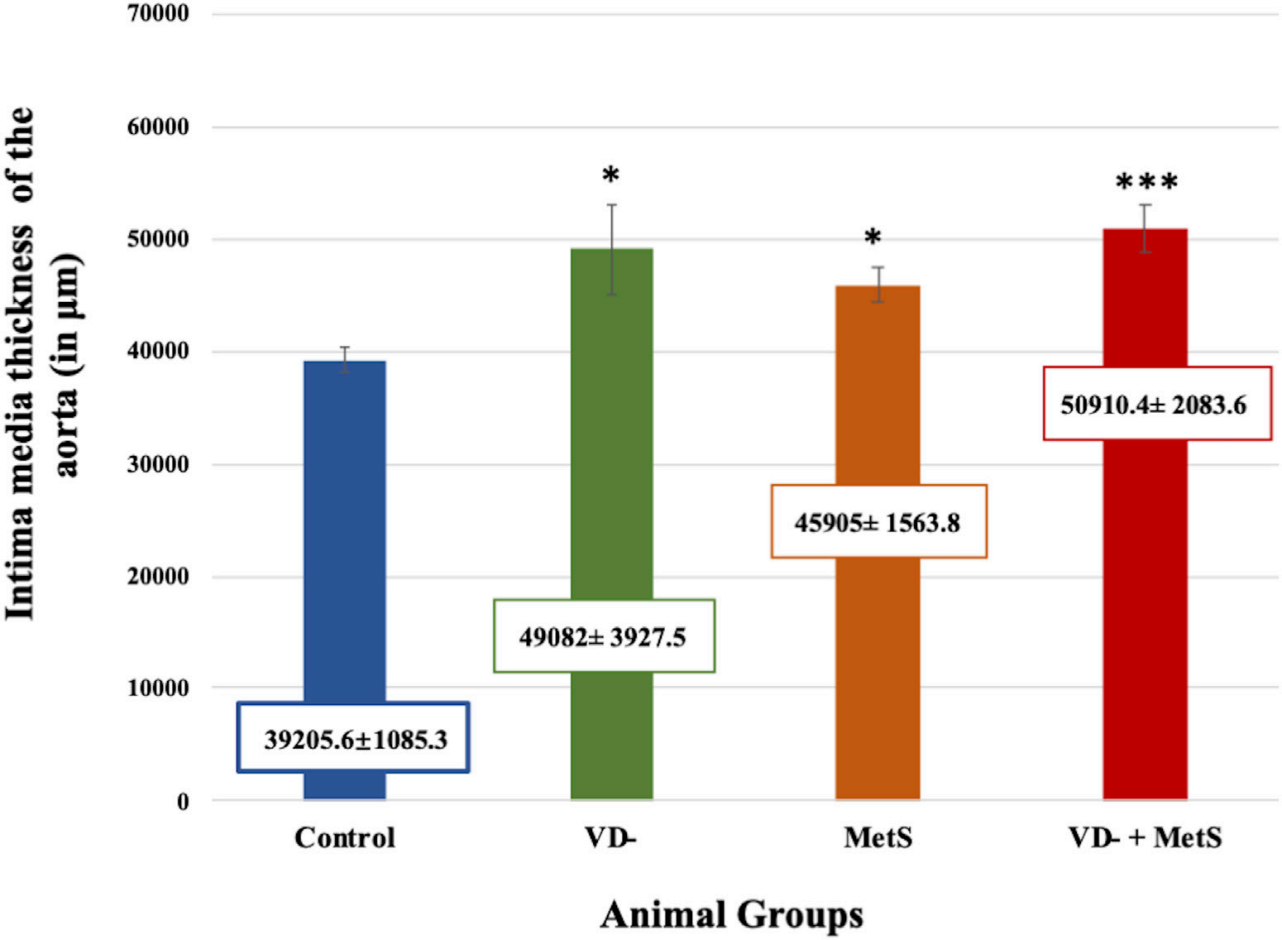
Mean ± S.E level of aortic intima-media thickness for control, VD-, MetS, and (VD- + MetS) groups after 8 weeks. VD-: vitamin D-deficient group, MetS: metabolic syndrome group, and VD- + MetS: combined group (vitamin D-deficient and metabolic syndrome), n = 12. Mean is expressed as mean ± SE. *** significant (p < 0.0001) vs control group. * significant (p < 0.05) vs. control group.

Aortic intima-media thickness was notably elevated in the VD- (p < 0.05), MetS (p < 0.05), and combined (VD- + MetS) groups (p < 0.0001) compared to control, with the highest values seen in the combined group, as shown in Figure 9.

## 4 Discussion

In this study, we emphasize the significant cardiovascular impacts of both vitamin D deficiency and metabolic syndrome (MetS), especially in instances where these conditions are present collectively. Our findings are consistent with and build upon prior studies, demonstrating the amplified impact of such disorders on cardiovascular remodeling, such as left ventricular hypertrophy, coronary artery thickness, and vascular dysfunction.

Metabolic syndrome, a well-known risk factor for atherosclerosis and heart failure, continues to be closely associated with poor cardiovascular outcomes (Grundy et al., 2005; Low Wang et al., 2016). In our study, the MetS group exhibited mild coronary artery thickening and left ventricular hypertrophy, highlighting the increased cardiac workload caused by hypertension, hyperglycemia, and dyslipidemia. Recent studies reinforce that metabolic syndrome induces chronic low-grade inflammation, which drives vascular remodeling and accelerates atherogenesis (Hlyan et al., 2024; Roberts et al., 2013). Moreover, recent findings suggest that insulin resistance in MetS increases oxidative stress and exacerbates vascular smooth muscle cell proliferation, which is consistent with the structural changes we observed in the coronary arteries (Morgado F et al., 2024). Elevated levels of CK-MB and LDH in the MetS group further indicate subclinical myocardial damage, which is consistent with recent reports that individuals with MetS are at increased risk of diastolic dysfunction and left ventricular hypertrophy even in the absence of overt heart disease (Chen et al., 2023).

Recent studies highlight the complex role of vitamin D in supporting cardiovascular health. Its deficiency is recognized as a significant factor in endothelial dysfunction, arterial stiffness, and cardiomyocyte apoptosis, thereby elevating the risk of heart disease (Barbarawi et al., 2019). This study reveals significant left ventricular hypertrophy and coronary artery thickening in the vitamin D-deficient group, aligning with recent findings that vitamin D functions as a cardioprotective agent through modulation of the RAAS and enhancement of endothelial NO synthesis (Chen et al., 2023). A 2020 meta-analysis demonstrated that vitamin D supplementation significantly enhanced vascular endothelial function, especially in individuals with low baseline vitamin D levels (Beveridge et al., 2018). Moreover, emerging evidence underscores the critical role of vitamin D in mitigating oxidative stress, regulating vascular tone, and preventing myocardial remodeling, which are key mechanisms in cardiovascular diseases (Islam et al., 2024). This study elucidates the moderate cardiovascular remodeling noted in the present study in the vitamin D-deficient group, characterized by endothelial dysfunction resulting in smooth muscle proliferation, evident in the thickened coronary arteries and ventricular walls. Our findings indicate that elevated CK-MB and LDH levels in the VD- group are associated with a heightened risk of myocardial infarction and heart failure observed in populations deficient in vitamin D (Cosentino et al., 2021). The assessment of metabolic syndrome parameters is not shown as it was previously published (Mahjoub et al., 2022).

The most significant cardiovascular changes in our study were observed in the combined vitamin D-deficient and MetS group (VD- + MetS), which exhibited severe left ventricular hypertrophy, coronary artery thickening, and vascular remodeling. Recent studies suggest that vitamin D deficiency worsens the metabolic profile in individuals with MetS, exacerbating inflammation, insulin resistance, and dyslipidemia, which synergistically drive cardiovascular pathology (Contreras-Bolívar V et al., 2021; Wang et al., 2016). A study by Lee and Kim (2021) demonstrated that individuals with both vitamin D deficiency and MetS had a significantly higher risk of cardiovascular events than those with either condition alone. This aligns with our findings that the VD- + MetS group exhibited the most severe vascular congestion, intima-media thickening, and smooth muscle proliferation, indicating that the synergistic effect of these conditions accelerates vascular aging and myocardial degeneration. Similarly, a recent study highlights the intricate interplay of genetic predisposition, molecular pathways, and structural changes in the aortic wall that contribute to vascular remodeling and degeneration (Godugu et al., 2024; Islam et al., 2023). It underscores the importance of early detection and innovative therapies to address vascular complications, which are particularly relevant in conditions such as those observed in the VD- + MetS group.

Furthermore, the increased oxidative stress observed in the combined group, as evidenced by elevated MDA levels, reflects the compounded effects of hyperglycemia, insulin resistance, and vitamin D deficiency on lipid peroxidation and vascular inflammation (Mahjoub and Masrour-Roudsari, 2012). The histopathological evidence of severe coronary artery remodeling and myocardial atrophy underscores the critical need for early intervention in individuals with both metabolic syndrome and vitamin D deficiency.

This study has several limitations that should be acknowledged. First, the study duration of 8 weeks may not fully capture the long-term effects of vitamin D deficiency and metabolic syndrome on cardiovascular health. Second, environmental and genetic factors that may influence the progression of metabolic syndrome and vitamin D deficiency were not explored in this study. Last, although the study identified key biochemical and histopathological changes, it did not investigate the molecular mechanisms in detail, such as specific signaling pathways involved in oxidative stress and vascular remodeling. These limitations highlight the need for further research to address these gaps and provide a more comprehensive understanding of the interplay between vitamin D deficiency and metabolic syndrome.

## 5 Conclusion

The present study provides robust evidence that both vitamin D deficiency and metabolic syndrome independently contribute to cardiovascular pathology, and their coexistence has a synergistic effect, exacerbating vascular and myocardial remodeling. These findings align with the recent literature from 2015 to 2024, which emphasizes the critical role of vitamin D in maintaining cardiovascular health and highlights the heightened cardiovascular risk posed by metabolic syndrome. Vitamin D supplementation, combined with effective management of metabolic syndrome, may be an essential strategy to reduce the cardiovascular burden in these populations. Future research should focus on studying the underlying mechanisms and molecular pathways of vitamin D in the progression of cardiovascular metabolic syndrome.

## Data Availability

The raw data supporting the conclusions of this article will be made available by the authors, without undue reservation.
